# Philadelphia Obstetrical Society

**Published:** 1890-03

**Authors:** 


### PHILADELPHIA OBSTETRICAL SOCIETY.

THURSDAY, JANUARY 2, 1890.

Dr. Theophilus Parvin in the chair.

AUGUSTIN H. GOELET.—The conservative treatment of inflammatory diseases of the uterine appendages and sequelæ by electricity. (See page 20.)

DR. G. BETTON MASSEY.—The treatment of chronic catarrhal salpingitis by electricity.

A student of gynecological literature in the future will doubtless regard with wonder a most singular epidemic that prevailed during the latter half of the eighth decade of this century. He will learn by a reference to the reports of the medical societies that the chief feature of this epidemic was a pus-tube, a thing that was regarded by the surgeons of the day as so dangerous that nothing short of amputation was to be thought of for a moment. If this student of history be at all philosophical, or even conversant with the medical fads and fashions of the past, he will no doubt regard this epidemic as of a subjective rather than an objective nature,—as a disease of doctors rather than of patients.

In making this statement I do not wish to deny the existence and frequency of salpingitis. It is with the views and practices that class this disease as practically malignant that I take issue. With or without a septic ætiology or the gonococcus, an inflammation of the lining membrane of the Fallopian tubes is simply a catarrhal inflammation of a mucous tract. It is an inflammation that does not differ materially from similar processes in other mucous cavities, such as the nose, the bronchi, the epididymis, or even that involved in chronic diarrhœa. That the whole of a tract is apt to be simultaneously affected is also too often lost sight of in this particular instance, the association of an endometritis with the salpingitis being, in my experience, universal. In the more chronic variety of the affection, to which my remarks will be confined this evening, the evidences of the uterine catarrh are usually present, though the disease may be latent in the uterine portion of the tract, just as similar evidences of a general rhinitis may be latent during the persistence of a post-nasal catarrh.

I allude thus briefly to the pathological nature of this affection in order to point out what I regard as a most important practical truth; namely, that to cure this affection we must first cure the causative endometritis in a large proportion of cases. The practice of ten years ago was distinctly in this direction, even if we did not then clearly understand the peri-uterine inflammation associated with the endometritis. Those old-fashioned applications of caustics and alterative solutions must have done some good in their day, or my respected teachers would not have used them so often as they did. That they did harm at times is equally certain; but with the clearer light of to-day, we can easily put our finger on the reasons. To begin with, they were often made with dirty instruments. That, in itself, was enough to cause frequent aggravations of the trouble to say the least. The second cause of failure was doubtless the severity and uncontrollable nature of the caustic agent. Finally, it is more than likely that a bulky, cotton covered applicator pushed forcibly to the fundus with the uterus steadied with a tenaculum has reversed the flow in many a case of this sort, forcing muco-purulent matter into the peritoneal cavity.

In a reaction from these methods of the recent past, the monstrous position has been taken by some of our younger abdominal surgeons that a muco-purulent catarrh of the tubes is essentially incurable, and that a woman so affected must suffer an instrumental removal of the diseased part at the risk of her life. That this rough cutting of the Gordian knot unsexes the poor creature, breaks up her home, and renders an eternal outcast among women is nothing to such enthusiasts, who, in heaping up a great laparotomy record, keep their operating bags packed for instant work, and do not hesitate to operate upon a woman the first time they see her, with or without the sanction of a professional consultation. Unfortunately the justification derived from relieving the pain and ill-health of those women who survive the operation is by no means assured. I have had an opportunity of following a number of these cases, in which removal of the appendages has been practiced for inflammatory disease, and I have yet to see one that was permanently benefited by it, most of them on the contrary being made worse in various ways. That many of them are relieved of pain during the period of enforced rest in bed incidental to the operation proves nothing. The after-results, in the shape of pain in the cut neural extremities, together with the sad effects upon the economy of the loss of so important a functional centre, more than counterbalance this temporary respite.

That this extreme act of surgery may be demanded as a palliative after the failure of conservative treatment is freely conceded; but it is maintained that the chief aim of the physician should be the restoration of the normal condition in these organs.

A plan of treatment that I have found highly satisfactory in chronic metro-salpingitis consists in a series of circumspect, carefully made galvanic applications to the endometrium, preceded and accompanied by vagino-abdominal applications of the same current, associated with rest and regulation of the alimentary tract; such a plan, particularly when carried out in a properly appointed institution or private hospital, will cure a large proportion of these cases. It will take time, of course, but a woman's health, and especially her womanhood, are worth time.

Taking up the parts of this plan of treatment in a reverse order of their importance, it may be claimed that mere purgation will relieve such cases. This is true in a limited extent in dispensary practice, but very rarely so among the more intelligent classes of ladies. Rest is recommended only as an adjunct to the more important portion of the electrical treatment, each intra-uterine application being most effective when performed at the patient's bedside and followed by absolute rest for twenty-four hours. The vaginal galvanic applications, a course of which should precede the applications to the cavity, are made every other day, the negative pole being applied by means of a large ball-electrode to that portion of the vaginal vault nearest to the tube or tubes, the indifferent pole being on the abdomen. The strength of the vaginal applications should vary from thirty-five to seventy-five milliampères, *pro re nata.* The intra-uterine applications, which I regard as the most important whenever an evident endometritis or chronic metritis co-exists, require also the most skill, since in addition to gentleness and cleanliness of the electrode management, both of which are essential to success, a delecate judgment must be exercised in arranging the frequency and current strength, and in desisting from this part if the conditions present are acute. I use currents for this purpose ranging from twenty to seventy-five milliampères, more usually twenty-five to forty, the pole selected depending on the presence of excessive or deficient menstrual flow. The frequency of the intra-uterine applications is about once in five days.

As an instance of what is possible from this treatment, I may cite the case of Mrs. D., aged 28, who had been ill since her only confinement, five years before first seeing me. This confinement was followed by sharp pains in both ovarian regions, irregular menstruation, and sterility. When first examined there was firm fixation of uterus, fulness in regions of both broad ligaments, and a slight uterine leucorrhœa, these conditions being verified by Dr. Constantine Goodell, who saw her with me at the Howard Hospital. Negative vaginal applications were begun (thirty to forty milliampères), the electrodes being forcibly pressed against the boggy masses. After the second and third applications there was an increased, intermittent leucorrhœal flow, accompanied by cramps. Examination now showed greater freedom of movement and less bogginess. A thirty-five milliampère negative application to the cavity was now made, and repeated five days later, followed by a normal menstruation, unaccompanied by the pains and dizziness of the preceding five years. After two more vaginal and one intra-uterine application there was a profuse yellow discharge, sudden and abundant, accompanied by cramps, evidently a discharge from tubes that had become patulous. The patient was immediately restored to health, and is now pregnant, having become so less than two months after the last application. Duration of treatment one month.

A similar case was that of Mrs. S. J., aged 32, who had not been well since the birth of her last child, one year before being seen first. She had had four children and three miscarriages, and had been suffering greatly from bearing-down pain and backache, and a voluminous leucorrhœa of a tenacious nature. Examination showed perinæum intact, uterus well forward and adherent to a boggy mass in the left tubal region, which was very tender. She recovered completely after eight vaginal negative applications of fifty and sixty milliampères. Duration of treatment, three weeks during August, 1889. She is now (Jan. 2, 1890) over three months pregnant.

The only other case that I will mention is that of Mrs. McE., aged 31, who had been ill seven years, and was the most obstinate of a number seen at the Howard Hospital clinic. She was an exceedingly delicate and nervous woman, the mother of four children, and had had seven or eight miscarriages, the last one two years before applying for treatment. For seven years she had been suffering from continuous shooting pains and tenderness in the left ovarian region, with a yellow, intermittent leucorrhœa, and a menstrual flow every two weeks. Dyspareunia intense and nervous system prostrated.

Examination showed perinæum intact, uterus enlarged and displaced to right, with a bilateral laceration of os. To the left of the uterus there was an exceedingly tender mass, within which a thickened tube and enlarged ovary was made out, the thinness of the patient adding materially to the ease of diagnosis. She had declined operation on a number of occasions, and various modes of treatment had failed.

Treatment was begun by the use of negative vaginal applications, thirty-five to fifty milliampères for three minutes. After the sixth vaginal and first intra-uterine application, she had a free discharge of pus, giving great relief. The case is still improving under a somewhat desultory treatment, her present condition being one of vast improvement in general health, with almost complete disappearance of the local morbid conditions, and a return of the periods to their normal frequency. Had this case been treated under more favorable circumstances, the duration of treatment would have doubtless been far less than the six months that was consumed.

All of these cases were what is known as operative cases; each had been advised to have the ovaries and tubes removed by one or more surgeons. I have related them in detail in order to prove that an occluded tube may be made patulous and capable of performing the duties assigned to it by nature.

DR. M. PRICE.—I have been much pleased with the paper of our friend from New York. Except in one or two instances he does not infringe upon the surgeons' field in abdominal surgery. I most certainly disagree in regard to electro-puncture. There are very few instances of tubal diseases in which the tube does not lie out of the reach of such puncture as he refers to. Again, the sacculated condition of the tube, and the associated conditions of the ovary and surrounding tissue, would usually render it impossible to reach and open the abscesses.

Another thing. In some four hundred cases, I have yet to see a case of tapped pyosalpinx or even hydrosalpinx, with very few exceptions, where the tube could float back to its normal position. On the contrary, it is often like quarrying out a cobble-stone from the pelvic wall or the abdominal wall. Their attachments may be almost anywhere. Occasionally they are in Douglass' pouch, and could be tapped if there were only one abscess. This, however, involves months of treatment. I have a case of pus-tubes which has been pronounced cured five different times in the last ten years. The last time I treated her found a ruptured tube, the belly full of pus, and the whole outlook was one of rapid death. There was no other treatment but removal. Our friend does not claim that these cases come within the bound of electricity, and any man that will operate for simple ovaritis, painful belly, or painful uterus, without being able to map out a positively diseased mass, is no true surgeon. The presence of pus and the temperature and pulse indicate that there is a condition which must be relieved—then, and then only, is the surgeon expected to operate. If electricity will do any good, I believe that it will do it in simple ovaritis and simple endometritis, if they exist, which I doubt.

Dr. Massey's onslaught on operators can be amply refuted by his own cases. I have seen cases he has pronounced well operated on by different men, and pus found. In Dr. Massey's cases, where there was recovery following the discharge of pus, it was not the effect of electricity, but simply good luck. Some years ago Dr. Keating saw with me a case in consultation. We decided that she had old-fashioned pelvic peritonitis, following labor. A few days later there was a profuse discharge of pus from the uterus. The woman is still living in moderately good health. She has been barren since. It was not our treatment that saved this case any more than it was Dr. Massey's treatment that saved his case. It was simply good luck.

Dr. J. M. BALDY.—In treating these cases of pelvic inflammatory diseases, electricians lose sight of the pathological condition; they treat the symptom (pain), but lose sight of the cause of the pain and the pelvic peritonitis. Pelvic inflammatory troubles can be divided into a great many forms. Pyosalpinx I shall not discuss, as electricity has absolutely no place in their treatment. We come then to that which is probably the most frequent and the most troublesome of the sequelæ of pelvic inflammations; that is, where the tubes and ovaries are simply adherent to surrounding parts. The tube and ovary may be healthy. At times the lining membrane of the tube is diseased, and the tube will roll between the fingers when removed like a piece of wire. Again, the tube may have undergone a cheesy degeneration. This is the class of which electricians make the most capital and in which they claim the most cures.

What, in such a case as this, does electricity do? Dr. Goelet, in a lecture before the polyclinic class in New York, stated that

"electricity melted adhesions like snow under the summer's sun." I have seen a good many cases in which there were adhesions (and in which I considered the adhesions the cause of the whole trouble) treated by electricity, and I have not yet seen a single adhesion melted, or a single case cured. There is one well-known case which has been often before this society, the specimen from which I hope to present to you before long, as she has requested me to operate. This case has been twice reported as cured, it has been reported in a notable work on electricity and in several journals as cured on as many different occasions. After being reported cured, the woman has in from one to two and a half months returned with another attack of pelvic peritonitis. She has now had some half a dozen such attacks while under treatment by electricity. That woman is in the same pathological condition as when I placed her in the hands of the electrician. The condition is simply one of adherent tubes and ovaries. These attacks of pelvic peritonitis were more quickly relieved by saline purges, tampons of glycerine, and other local measures, than they have been by electricity.

If in these cases where there are accumulations we puncture and drain, we leave just what we have been describing,—a diseased and adherent tube and ovary. Puncture removes the accumulation but does not cure the disease. The disease remaining, pain and peritonitis will surely occur periodically to a greater or less degree. So far as opening the uterine end of the tube by electricity and so draining it is concerned, I think that is nonsense. After removal of the tube, I have often tried to pass a sound or even a bristle through the uterine end of the tube and have uniformly failed. In regard to passing instruments into such a tube through the uterus, that seems the height of absurdity.

Dr. Massey claims that if treated in a hospital these cases may be cured. Not one in a hundred patients can afford to have treatment in a hospital long enough to be relieved, let alone cured; at any rate, they could not be permanently cured if they stayed there until doomsday.

DR. J. PRICE.—This subject requires plain speaking. I am something like the Sunday-school boy, who, after he had been told the Jonah story and then the Daniel story, said, "Hold up; you are going a little too far." I feel so about this electrical business. I am amazed at the claims of these two electrical papers. The subject scarcely belongs to an obstetrical and gynecological society. But there are a few points of which I shall speak. One of the gentlemen prefaces his paper with remarks on conservative gynecology, and speaks of sterility. I control probably the largest gynecological clinic in America. Hundreds of women come who have been sterile one to ten years, with evidences of slight mischief on one side or both sides,—the class of cases to which Dr. Massey refers. After receiving local applications, hot-water douches, etc., with general treatment, they conceive and bear children. I wish to call attention to my experience in the Midnight Mission, an institution for reclaiming fallen women. For a long time I was physician to that institution, and my experience there shows what rest for six months and general treatment, without local treatment, will do for sterility. Some of these women had had one child and been sterile for five or six years, although they had many opportunities for becoming pregnant. Many of these women after leaving the institution restored in general health became pregnant in a few months. One speaker alludes to pain following operations. There are a few operations that should not be done; but if he is alluding to operators who are doing good legitimate work in Philadelphia, he is mistaken. I have here a bucketful of pus-tubes, some as large as sweet patatoes, to demonstrate my position. I would sooner think of cutting off my right hand than of removing an ovary for ovaralgia, backache or hysteria. I have frequently been asked to remove the ovaries in such cases, but have always declined.

Chronic endometritis is not so common among gynecologists as it is among electricians.

I specially ask these elctricians why it is that suppurating glands in the groin, neck, popliteal abscess, palmar abscess, and pus in other parts of the body receive no attention at his hands, while pus in the pelvis, the existence of which he guesses at, under the influence of electricity melts like "dew before the summer's sun?" They have a beautiful classification of cases into the first, second, and third stages. I have called attention to the so-called "acute" stage a few minutes ago, where, under rest, arsenic, iron, strychnia, fresh air and good diet, the women have conceived. These cases, the specimens of which I present, had lost flesh, had quick pulse and a high temperature. Some were unable to walk. Many of them had refused operation and undergone a variety of treatments, electrical and other. You put them to bed, purge them and prepare them for operation, and in two or three days they are free of pain and feel so well that occasionally some refuse operation. Here is a typical pus-tube. That tube was six inches long. I show you two pus-pockets,—what would the electrician do with these cysts and multiple pus-sacs? These cases are always complicated. Everything is involved, from the promontory of the sacrum to the pubic bone, and from vermiform to sigmoid.

Here is another tube into which it would have been impossible to pass a filiform bougie. This tube contained seven pus-sacs as large as hickory-nuts and walnuts, with an ovarian abscess and a small broad-ligament cyst. On the other side there were some five pus-sacs, making in all about thirteen abscesses. It would have required some skilful work to have tapped all of these with these little electrie trocars.

These are all pus-tubes. Some of the women have refused operation, and later asked for it. These operations have not been done simply for the relief of pain. Of course it is our duty to relieve suffering. There is nothing that makes me prouder of my worth than the fact that many of the women on whom I have operated for pus are now among the healthiest and most energetic women walking the streets of Philadelphia.

Dr. Massey claims complete cures. They have never proven their case. Could I open the abdomen and display a single or double pyosalpinx or hydrosalpinx, and without removing them introduce two or three stitches, I would gladly turn one of these cases over to the electrician and let him demonstrate how he could open the tube, make the fimbriated extremity stand out, and the women conceive at the first opportunity. I am amazed to hear of such marvelous cures without sufficient data being given to fortify the statements made. I sometimes feel it is almost criminal to attempt these cases without accurate diagnosis. It looks like simply extorting money from these patients. I often wonder why electricians have given up the general application of electricity. The electrical expert in gynecology was a short time ago making general applications of electricity for nervous disturbances. It would be of value if they would tell us what they accomplished by general application of electricity, why they gave it up, and why, without special training in gynecology and the treatment of pelvic troubles, they take it up and pose as experts. I feel that no man knows anything about pelvic inflammatory troubles until he has dealt with them both from above and below. They want to study their pathology, and not to give us the pathology of forty years ago. We are too far from Egypt and the pyramids to plough our ground with crooked sticks.

DR. AUGUSTIN H. GOELET, New York, in closing the discussion said: It seems to me unnecessary to make such a tirade about pus-tubes. I stated in the paper that I limited the aspiration to certain conditions. I operate myself for tubes that cannot be cured. There are many cases, however, that get well even without electricity. They conceive and enjoy good health afterwards. The fact that they are able to conceive I know does not show positively that they are well; but this would be impossible if laparotomy were performed; for if we found one tube diseased and the other suspicious, both would be removed. I do not see the necessity of the exhibition of these specimens. I do not say that a single one was removed unnecessarily, if it was done to save life. I have, however, cured many of these cases with electricity, and a number of the women have become pregnant and enjoyed good health afterwards, and, so far as it is possible to determine by palpation, they are anatomically cured. I can relate one case which I aspirated shortly after my return from Europe. She came to my office with a distended tube in the cul-de-sac. I sent her home and told her to clear out the bowels. I examined her two days later and found the tube greatly distended. I tapped the tube through the vagina and withdrew an ounce and a half of muco-pus. The patient is well to-day with no evidence of disease, not even a thickened condition of the tube.

Those who say that salpingitis cannot be cured by electricity are mistaken and do not know what they are talking about. I was a gynæcologist before I began the use of electricity, and I do not claim to be an electrician. Hypertrophy can be cured by electricity. I have said that exudations and adhesions will disappear rapidly under the influence of strong galvanism, but do not claim that all adhesions can be removed. The fimbriated extremity of the tube when adherent may not be forced up into position by electricity; but if the distended tube is emptied it will assume somewhat its normal position. Then, by relieving the inflammatory condition at the proximal end and removing by absorption the exudation which surrounds it, drainage may take place through the tube into the uterus. The previous speaker has stated that many of the cases formerly considered to be endometritis were instances of mistaken diagnosis, and were really pyosalpinx, which is true. Such cases may and do recover under treatment by electricity when drainage through the uterus can be secured.

What became of these cases ten or fifteen years ago before the operation for removal was devised? Many of them got well even under the older methods of treatment. They were not operated upon then, and very few of them died. When a fluctuating tumor was found in the vagina it was opened as an abscess, and the patient got well. Now, what I advocate is emptying the abscess or distended tube in the vagina when it can be reached, and the use of the galvanic current through the aspirating needle after the removal of the pus, in order that there may be no extravasation into the surrounding tissues.

The argument in regard to abscess in other parts of the body is unfortunate for the gentleman who advanced it. He would not amputate the finger for a whitlow, or the head for an abscess of the neck, but he would let out the pus. This is just what I advocate.

There are many cases of pyosalpinx that do not demand immediate operation or amputation, and in some of them the pus can be reached by the vagina. I fancy that these gentlemen would not risk to have the testicles removed without some effort being first made to cure the disease.

All I advocate is that we try to cure some of these cases instead of operating hastily. Frequently operation is advised and practiced when unnecessary. I can recall a case which came under my observation, on which the operation was done simply for hemorrhage from the uterus following miscarriage. The curette had been used with benefit, and in about a week the flow stopped. The abdomen was then opened and healthy tubes and ovaries removed. The patient has suffered pain ever since, or until she was relieved by bipolar faradization. She never had a pain before the operation.

There is a mistaken idea in regard to the applications of electricity to the tube. I do not try to make applications inside the tube. The applications are made to the endometrium, and the effect upon the tube is obtained by the current passing along the muscular structure. The galvano punctures are made into hypertropied tissue, and the current is carried into it by means of the needle. This method of treatment does cure many of those cases of fulness and thickening of the broad ligament which to-day are being submitted to operation for removal.

Dr. G. BETTON MASSEY.—I have but little to add to the reply made by Dr. Goelet. I was strong in my condemnation of hasty operation for these inflammatory conditions because I feel that we are drifting into a fashionable fad that on second thought would be abandoned to a certain extent, and because I had had positive results that make me capable of speaking on the matter. Dr. Price misconceives my ideas entirely in regard to the cure of pus-tubes, and in supposing that I catheterize the tube. My object in these applications to the endometrium is to bring about resolution in the mucous membrane, and as the mordid process went upwards, so the curative process may extend in the same direction.

The comparison in regard to inflammatory abscess in the neck and elsewhere is an unfortunate one. These conditions in the pelvis are not abscesses, according to the correct use of the word. They are collections of pus in a portion of a natural cavity. If they can be relieved by a correction of the morbid process that causes the accumulation, they should be.

What we want in a society like this is not the exhibition of a bucketful of specimens, but a careful statement of the results a year after treatment. I do not say that the results after operation will not be good, although they have not been in the cases seen by me; but the whole facts should be given to us. It should be remembered that this operation is a new thing and is still on trial.

To illustrate what is thought of it by some gynecologists I will quote two sentences from a letter by a prominent gynecologist of New York, Dr. Thomas Addis Emmett, which appeared in a recent number of the *New York Medical Journal.* "The burden of proof must rest with those who claim so much for the operation, to show that the after-condition of their patients justifies its frequent performance. If they neglect to vindicate themselves, the verdict must be, if it rests on the judgment of others, that while it may prove a source of pecuniary profit to the operator, a very large number of women who trust to their honesty have not been benefited, and their condition has been made a more deplorable one."

In reference to Dr. Baldy's remarks on a case that has not been permanently cured, and which I have referred to him for operation, he should remember that the conditions are entirely different from those under discussion, a prolapsed ovary being the most prominent feature. This is, moreover, a clinic case that will not observe the personal hygiene during treatment which might have changed her six months' relief into a permanent cure.

The cases I have cited prove that diseased tubes may be so completely cured as to be able to perform their highest normal function,—that associated with pregnancy; and it follows that no operation involving a permanent destruction of the parts should be performed until this method has been intelligently and skilfully tried. To say, as some have this evening, that pregnancy occurs anyway in these cases, is absurd. If these gentlemen think that pregnancy is possible, why do they perform this operation?

Adjourned.

